# The Comparative Effectiveness of Rodents and Dung Beetles as Local Seed Dispersers in Mediterranean Oak Forests

**DOI:** 10.1371/journal.pone.0077197

**Published:** 2013-10-23

**Authors:** Ignacio M. Pérez-Ramos, José R. Verdú, Catherine Numa, Teodoro Marañón, Jorge M. Lobo

**Affiliations:** 1 Instituto de Recursos Naturales y Agrobiología de Sevilla (IRNAS), Consejo Superior de Investigaciones Científicas (CSIC), Sevilla, Spain; 2 I.U.I Centro Iberoamericano de la Biodiversidad, Universidad de Alicante, San Vicente del Raspeig, Alicante, Spain; 3 Dep. Biogeografía y Cambio Global, Museo Nacional de Ciencias Naturales, Consejo Superior de Investigaciones Científicas (CSIC), Madrid, Spain; University of San Diego, United States of America

## Abstract

The process of seed dispersal of many animal-dispersed plants is frequently mediated by a small set of biotic agents. However, the contribution that each of these dispersers makes to the overall recruitment may differ largely, with important ecological and management implications for the population viability and dynamics of the species implied in these interactions. In this paper, we compared the relative contribution of two local guilds of scatter-hoarding animals with contrasting metabolic requirements and foraging behaviours (rodents and dung beetles) to the overall recruitment of two *Quercus* species co-occurring in the forests of southern Spain. For this purpose, we considered not only the quantity of dispersed seeds but also the quality of the seed dispersal process. The suitability for recruitment of the microhabitats where the seeds were deposited was evaluated in a multi-stage demographic approach. The highest rates of seed handling and predation occurred in those microhabitats located under shrubs, mostly due to the foraging activity of rodents. However, the probability of a seed being successfully cached was higher in microhabitats located beneath a tree canopy as a result of the feeding behaviour of beetles. Rodents and beetles showed remarkable differences in their effectiveness as local acorn dispersers. Quantitatively, rodents were much more important than beetles because they dispersed the vast majority of acorns. However, they were qualitatively less effective because they consumed a high proportion of them (over 95%), and seeds were mostly dispersed under shrubs, a less suitable microhabitat for short-term recruitment of the two oak species. Our findings demonstrate that certain species of dung beetles (such as *Thorectes lusitanicus*), despite being quantitatively less important than rodents, can act as effective local seed dispersers of Mediterranean oak species. Changes in the abundance of beetle populations could thus have profound implications for oak recruitment and community dynamics.

## Introduction

Plant-animal interactions are key components in the regeneration process of many plant species with strong implications for population viability and community dynamics [1]–[2]. Seed dispersal, which connects the end of the reproductive cycle of adults with the recruitment of their offspring [3], is frequently mediated by biotic agents [4]. There is growing evidence that the number of new adults produced by the activity of a disperser (seed dispersal effectiveness, or SDE) not only depends on the quantity of dispersed seeds but also on the quality of the seed dispersal process [5]. In turn, the qualitative component of SDE depends on other factors such as the probability that a dispersed seed remains viable after the activity of the dispersal agent or the recruitment suitability of the microhabitat where the seed is deposited [6]. However, the quality of seed dispersal has received far less attention than the quantitative component, likely due to the methodological complexity entailed in their measurement. First, an accurate evaluation of dispersal quality implies an exhaustive relocation and monitoring of the dispersed seeds in order to determine the seed fate. Second, a complete determination of the microhabitat suitability for recruitment requires a multi-stage demographic approach where plant recruitment is explored across multiple and consecutive stages [7]. Nevertheless, most studies linking seed dispersal and plant demography have focused on early demographic processes, such as seedling emergence and first-year survival, often ignoring the delayed consequences of seed deposition at later stages of recruitment [8]. Since the suitability for recruitment of different microhabitats may change over the life cycle of the plant [9]–[10], studies based only on early life-history stages can lead to incomplete and misleading conclusions.

Recent studies have shown that both the quantity and the quality of seed dispersal are highly context-dependent, highlighting habitat structure as one of the main drivers of the spatiotemporal variation in SDE [6]. On the one hand, the foraging pattern of dispersers is not random, usually responding to preferences for certain habitats or microhabitats and rejection of others [8], [11]. On the other hand, the spatial distribution of seed deposition across different microhabitats will alter the subsequent stage-specific probabilities of recruitment [1], [12]. Empirical studies on SDE explicitly considering the heterogeneity of habitat structure are therefore necessary for better understanding the role of this source of variation in plant-animal interactions.

In most cases, the seed dispersal of animal-dispersed plants is due to a small set of biotic agents [5]. However, the contribution that each of these dispersers makes to overall plant recruitment may differ largely [13]–[14]. Broad differences in effectiveness among dispersers may have important ecological and evolutionary consequences for the plant as well as profound implications for the conservation of the most effective dispersers [15]–[16]. However, comparisons among seed dispersers in SDE have seldom been explored [13]–[14], [17], especially in scatter-hoarding animals. In the specific case of temperate tree species with large-seeded dry fruits such as oaks, jays and rodents (such as those belonging to the genera *Apodemus, Mus, Sciurus*, etc.) are broadly considered the main guilds of seed dispersers worldwide [18]. Nevertheless, a novel and striking feeding behaviour has been recently described for some species of geotrupid dung beetles inhabiting Ibero-Mediterranean [19]–[20], and North American oak forests [21]. These species of dung beetles are able to bury and feed on acorns, and this rare habit confers on them important ecophysiological advantages, such as a significant increase in their ovarian development, thermal tolerance and pathogen resistance [22]–[23]. This burying behaviour could also bring reproductive benefits to the oaks because a portion of the buried acorns could be abandoned partially eaten or even intact (i.e., with the embryo undamaged) with the ability to emerge and become established as seedlings.

In this paper, we compared for the first time the relative contribution of two local guilds of scatter-hoarding animals (rodents and dung beetles) with contrasting foraging behaviours and metabolic requirements (rodents are basically endothermic organisms capable of maintaining stable internal body temperatures, whereas beetles are ectotherms in which internal physiological sources of heat are relatively small or quite negligible) to the overall short-term recruitment of two oak species co-occurring in the forests of southern Spain. Despite the unquestioned role of jays for oak regeneration at large distances [24]–[25], we selected rodents for this comparative study because of their potentially redundant role with beetles and their quantitative importance as acorn dispersers at small spatial scales [18].

We used a multi-stage demographic approach (from seed dispersal to third-year seedling survival) and followed the framework described by Schupp *et al.* [6] for disentangling the relative importance of the quantitative and qualitative components of SDE. Seed dispersal and recruitment were explored along a wide and natural range of microhabitat conditions, which allowed us to incorporate the role of forest habitat heterogeneity in order to evaluate the effectiveness of local acorns dispersers on a spatial scale. Specifically, we aimed to answer the following questions: (i) What is the comparative effectiveness of rodents and dung beetles as local acorn dispersers of oak species in a Mediterranean forest of southern Spain?; (ii) Which one of the two SDE components (quantity or quality) better contributes to overall short-term recruitment of these oak species?; (iii) How do these plant-animal interactions change with microhabitat conditions?; and (iv) What are the ecological and management implications of these findings for forest stand dynamics and species conservation?

## Methods

### Ethics Statement

All necessary permits were obtained for the field studies described herein thanks to J. Manuel Fornell Fernández, Director of Los Alcornocales Natural Park.

### Study Area and Species

The study was conducted in La Sauceda forest (530 m above sea level, 36°31′54″N, 5°34′29″W), located in the mixed-oak forests of the Aljibe Mountains in southern Spain. The climate is subhumid Mediterranean, with mild and wet winters alternating with hot and dry summers. Annual mean temperature ranges from 14.6 to 18.4°C (mean of 17°C). Annual mean rainfall varies from 900 to 1800 mm (mean of 1265 mm). Vegetation is dominated by evergreen cork oak (*Quercus suber*) forests mixed with winter-deciduous oaks (*Q. canariensis*), which are more abundant near streams [26]. The shrubby understory is diverse and rich in endemic taxa [27]. Most of the forested area was protected in 1989 as Los Alcornocales Natural Park, covering approximately 1680 km^2^.

The two dominant oak species show a strong inter-annual variability in seed production [28]. Acorn production was relatively low during the two sampling periods (2009/2010 and 2010/2011 cycles). The estimated acorn crop size for the two sampling periods was, respectively, 7 and 10.8 g m^−2^ y^−1^ beneath mature trees of *Q. suber* and 22.4 and 14.7 g m^−2^ y^−1^ beneath *Q. canariensis.* Acorns mainly fall to the ground during autumn and are potentially dispersed by animals: over large distances by jays and locally by rodents and dung beetles (in particular *Thorectes lusitanicus* [19]–[20]). *Thorectes lusitanicus* Jeckel (Coleoptera, Scarabaeoidea, Geotrupidae) is a flightless, medium-sized species of dung beetle (mean dry body weight of 130–175 mg specimen), endemic to the southern Iberian Peninsula [29]. This species is classified as a ‘telephagic tunneller’ which has acquired the ability to ship dung from its source to the nesting site, occasionally several metres distant, and bury it up to 10–15 cm deep. Recent studies have shown that this burying behaviour is also followed when they manipulate acorns [19]–[20]. Large herbivores, such as red deer *Cervus elaphus*, roe deer *Capreolus capreolus* and free-ranging cattle are abundant in these forests and could act as potential acorn predators.

### Sampling Design and Data Collection

We collected acorns of *Q. suber* and *Q. canariensis* in the study area from several trees (at least ten of each species) to encompass intra-specific variation. We selected healthy medium-sized acorns, discarding those infested by moth or beetle larvae through flotation [30]. The selected acorns were stored on a moist substrate at 2–4°C until used in the experiments and individually weighed to the nearest 0.01 g. The mean ± SD (standard deviation) acorn fresh weight was 5.37±1.70 g for *Q. suber* and 5.81±1.52 g for *Q. canariensis*. Acorns of known weight were further distributed in 54 experimental units (a minimum distance of 25 m relative to one another) and equally separated into three types of microhabitat, which spanned a wide gradient of plant cover and light availability: (i) inside dense shrub and tree overstory; (ii) under oak trees without shrub understory; and (iii) open microsites. In general, these three types of microhabitats appear intermingled in the understory forming a mosaic of different microsites separated among them by similar distances. Open areas tend to suffer events of sporadic soil waterlogging during the wet period due to less interception of rain by the canopy and, in most cases, a higher proportion of clay [31]. The shrubby vegetation comprised mainly *Pistacia lentiscus, Phillyrea latifolia, Viburnum tinus, Erica arborea* and *Erica scoparia*.

#### Seed handling

To explore spatiotemporal patterns of seed handling by local dispersers, we individually numbered with a permanent maker groups of 30 acorns (15 per oak species) and randomly placed them on the forest floor in each of the 54 above-mentioned experimental units (18 in each type of microhabitat). Acorns were protected from birds and large herbivores in each experimental unit by wire cages (80×80×25 cm) with 4-cm^2^ mesh that allowed the entrance of only rodents and beetles. In mid-autumn (November of 2009 and 2010), coinciding with the onset of the acorn-crop, we intermixed each group of 30 acorns and distributed them inside each cage in five equidistant lines of six acorns (three per oak species). We periodically checked all the experimental acorns during a whole reproductive-cycle (from December to October-November of the next year). To avoid interference with beetle foraging during the process of acorn consumption, we visited each of the five lines of six acorns once at different dates. The first line of acorns was checked 10–14 days after starting the experiment, coinciding with the peak of maximum beetle activity [19]; the remaining censuses were carried out at four regular intervals of time (approximately every two months). On each visit, we recorded the status of the acorns (intact, removed, or consumed *in situ*), noting when possible the identity of the dispersal agent (rodent or beetle). The acorns consumed *in situ* by rodents were easily identified by signs of gnawing, whereas those handled by beetles usually showed a circular (beetle-sized) hole in the coat. When we did not locate an experimental acorn in its original site, we dug 10–15 cm deep within a circle 50 cm in radius to verify evidence of handling by the dung beetle *Thorectes lusitanicus.* When a buried acorn was found intact, it was assigned to *T. lusitanicus* handling only when a beetle was also buried together with it.

#### Seed deposition and caching

To investigate patterns of seed deposition and caching, we carried out a parallel experiment with acorns freely exposed to all the animals (i.e., without the protection of cages) to avoid the interference of cages with the process of seed dispersal. In mid-autumn (November 2009 and 2010), we randomly distributed 54 additional groups of ten acorns (five per oak species) along the same above-mentioned experimental units to determine acorn fate (seed status and microhabitat destination). In each experimental unit, we placed five acorns (without cupule) per species on the ground, four in the corners and one in the centre of a 0.5-m^2^ quadrat. These acorns were individually weighed and attached to a metal wire (10 cm long by 0.6 mm wide) by drilling a hole with a needle. We marked both the metal wires and the corners of the quadrats with numbered coloured flags for monitoring acorn fates. We used this method of seed tracking (other examples in [32]–[33]) because it has been demonstrated that it does not significantly alter acorn dispersal patterns by small dispersers [34].

We periodically visited (at the same censuses specified above) all the experimental acorns and also classified them as intact, removed, or consumed *in situ.* We searched outwards for the removed acorns in the experimental units in expanding circles to a maximum distance of 30 m. We considered an acorn to be dispersed when it was moved horizontally and/or vertically (i.e., cached) from its original point by rodents or beetles. To characterise spatial patterns of seed dispersal and caching, we recorded the distance from the experimental unit and the type of microhabitat where the acorn was dispersed (i.e., microhabitat destination). We also noted whether the relocated acorn remained potentially viable (i.e., intact or partially consumed with the embryo undamaged) or not (i.e., completely or partially eaten with the embryo damaged, hereafter ‘preyed upon’). Those removed acorns that were not further relocated (i.e., missing acorns) could not be assigned to any of these two categories and were excluded from statistical analyses. Although we detected some evidence of seed re-dispersal over the sampling periods, we only considered the final fate of experimental acorns (i.e., seed status and microhabitat destination at the last census) for statistical analyses due to its higher relevance for recruitment.

We repeated the experiments of seed handling and seed deposition during two consecutive sampling years (2009/2010 and 2010/2011), making a total of 4320 experimental acorns (2160 per oak species) that were individually monitored to explore the spatiotemporal patterns of seed handling and dispersal.

#### Seedling recruitment

Microhabitat suitability for seedling recruitment (seed germination×seedling emergence×seedling survival for three years) was previously assessed by conducting a parallel seed-sowing experiment at the same study site where seed handling and dispersal were evaluated. In December 2003, we randomly placed a total of 1200 acorns (600 per oak species) across 60 experimental units evenly distributed along the same previously mentioned three types of microhabitats. We sowed ten acorns per species in each experimental unit at 1–3 cm depth, simulating biotic seed dispersal and burial by European jays [24]–[25], rodents [29] or dung beetles [19]. In this case, the experimental units were protected by smaller wire cages (25×25×25 cm and 1.3 cm mesh size) to exclude all potential acorn consumers. We indirectly assessed seed germination after the first summer by unearthing non-emerged seeds and inspecting for the presence of radicles. We periodically monitored seedling emergence and survival during three sampling years (2003/2004, 2004/2005 and 2005/2006). Censuses were carried out bi-weekly during the first year and every six months (in late-winter and after summer, coinciding with the peaks of maximum seedling mortality) during the two remaining years (see [32] for further details).

### Data Analyses

#### Spatiotemporal patterns of seed handling, caching and deposition

We first studied seed handling patterns for rodents and dung beetles using Generalized Linear Models (GLMs; [35]) and classifying the acorns in two possible states: intact *versus* handled (including both consumed *in situ* and removed acorns). We assumed a logit link function between this binomial dependent variable and two independent variables (oak species and microhabitat of origin). This analysis was applied separately for the five censuses carried out in each of the two sampling years.

Second, we examined seed viability in the experiments on seed deposition and caching using Generalized Linear Mixed Models (GLMM). The dependent variable was fitted to a multinomial distribution with four categories depending on the combination of the dispersal agent (rodents or beetles) and the final seed status (preyed versus undamaged). The explanatory variables in this case were the two sampling years (2009/2010 or 2010/2011), the oak species (*Q. suber* or *Q. canariensis*) and the three microhabitats of origin (shrub, tree or open). Seed mass was also included in the analyses as a continuous co-variable. The microhabitat distribution of dispersed acorns (i.e., microhabitat destination) was also studied using GLMM. In this case, the dependent variable was treated as a multinomial with three categories (shrub, tree or open), using the same explanatory factors described in the previous analyses. Seed dispersal distance (log-transformed) was fitted to a normal distribution and, therefore, the effects of the above-described factors were evaluated using Linear Mixed Models (LMM). In all these analyses, the experimental unit was included as a random factor.

Finally, microhabitat suitability for seedling establishment was assessed by testing the differences between microhabitats for the different stage-specific probabilities of recruitment considered in this study. Seed germination, seedling emergence and seedling survival were fitted to binomial distributions and analysed with Generalized Linear Models. GLMMs were performed using the lme4 package in R (v. 2.12.0, R Development Core Team, 2006). The remaining analyses were carried out using Statistica (v. 6, StatSoft Inc., 2001).

#### Seed dispersal effectiveness

Seed dispersal effectiveness (SDE) was calculated for each of the two scatter-hoarding animals considered in this study (rodents and beetles) as the proportion of seeds dispersed by each dispersal agent (i.e., quantity of dispersal) multiplied by the probability that a dispersed seed becomes established as a third-year seedling (i.e., quality of dispersal) (see a recent review [6]). The qualitative component of SDE was in turn separated into two subcomponents: the probability of a dispersed seed being cached and undamaged rather than preyed (i.e., seed viability) and the probability of overall recruitment in the microhabitat where the seed was transported (i.e., quality of seed deposition). Thus, we obtained six values of SDE (two dispersal agents×three types of microhabitats) for each oak species. In addition, we calculated a global value of SDE for *Quercus* grouping the two studied oak species.

## Results

### Seed Handling

**Figure 1:**
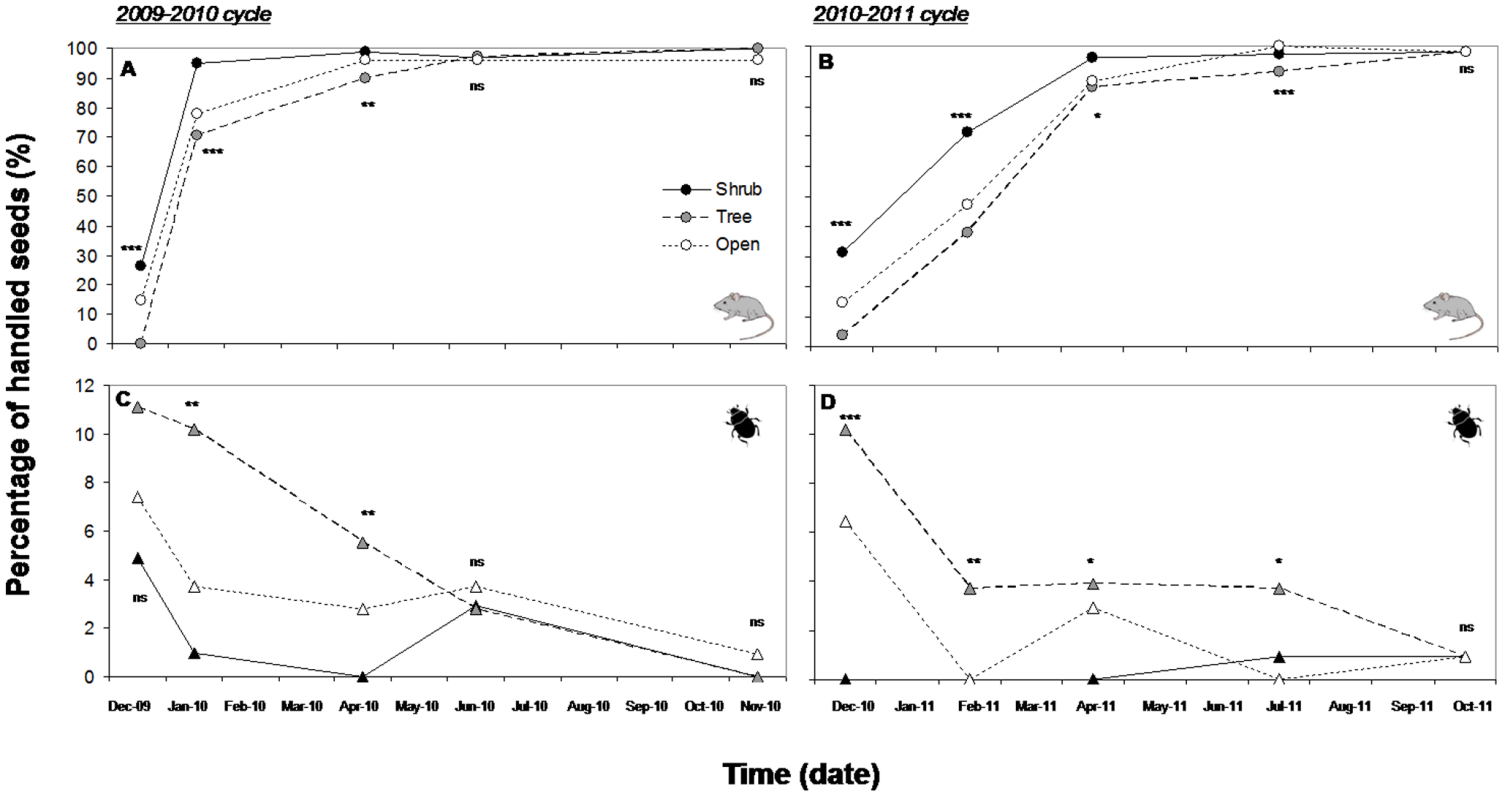
Temporal dynamics of seed handling by rodents and beetles during the two sampling years. Significant differences among the three types of microhabitat considered in this study are indicated as follows: *p<0.05; **p<0.01; ***p<0.001; ^ns^not significant.

The percentage of seeds handled by local dispersers was close to 100%, especially for the second sampling year (2010/2011; Fig. 1). These high rates of seed handling were mainly due to rodents, which reached values over 80% from mid-spring (Figs. 1A and 1B). A lower proportion of the experimental acorns (less than 11%) were handled by dung beetles (*Thorectes lusitanicus*), with the highest rates of handled seeds during the first census (December 2009 and 2010; Figs. 1C and 1D). Rodents and beetles showed contrasting differences in the spatial pattern of seed handling depending on the type of microhabitat where the seeds were experimentally placed. The percentage of seeds handled by rodents was significantly higher under shrubs compared with the other two types of microhabitats, although these differences disappeared as seed handling approached 100% (Figs. 1A and 1B). In contrast, beetles showed higher rates of seed handling under trees, followed by open sites and finally under shrubs (Figs. 1C and 1D). No statistically significant differences were found between the seeds of the two oak species (data not shown).

### Seed Deposition and Caching

**Figure 2:**
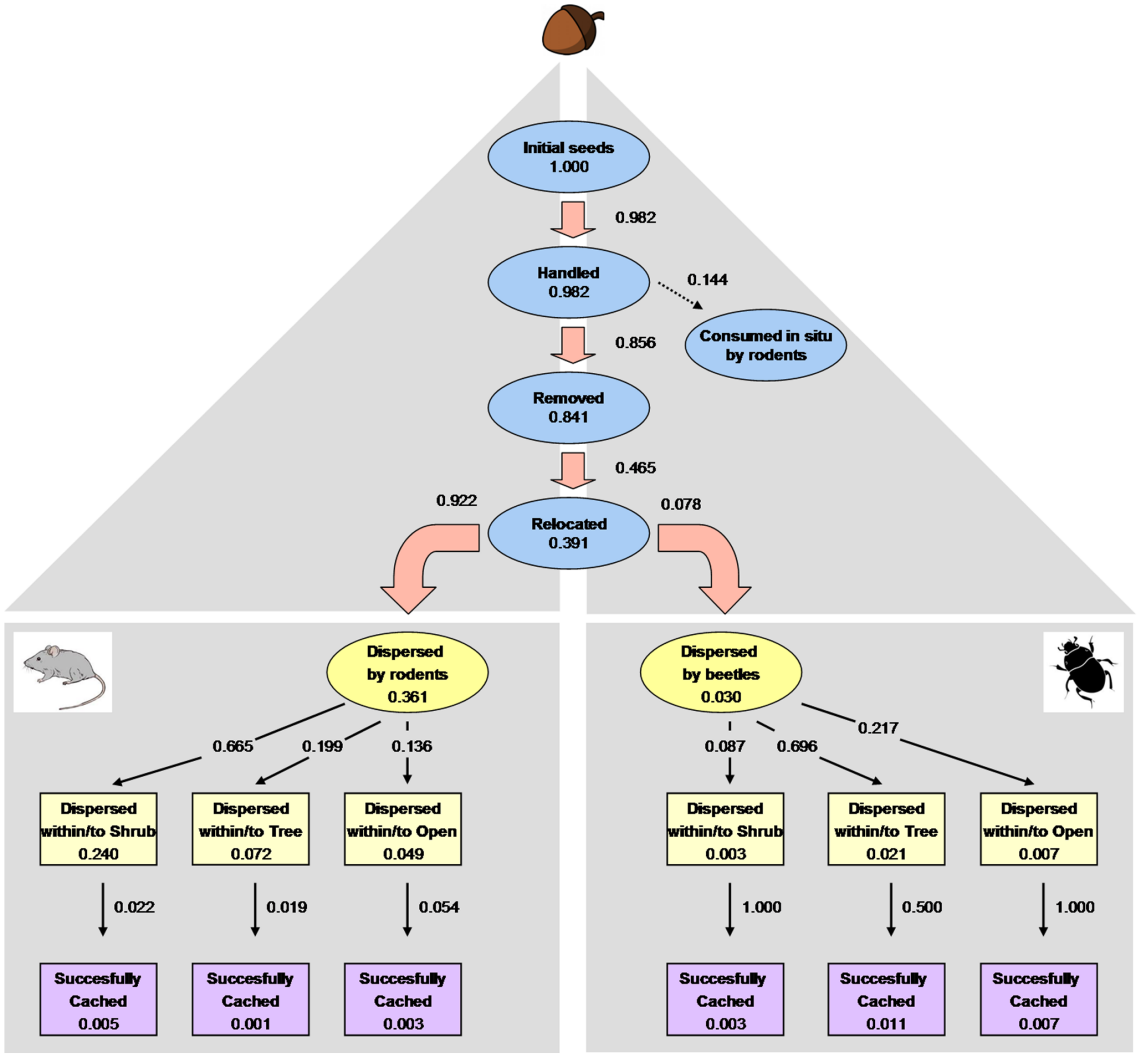
Summary diagram of acorn fate. The values next to the arrows indicate the transition probabilities among the different categories; the values inside boxes indicate the proportion of the initial experimental acorns (N = 1080) still alive at each of these categories. Transition probabilities were calculated by mixing the two sampling periods (2009/2010 and 2010/2011) and the two oak species (*Q. suber* and *Q. canariensis*). ‘Cached’ and ‘undamaged’ have been grouped into the same category because we did not find any case of removed and non-cached acorns that remained viable over the whole sampling period.

Results provided by the experiment on seed deposition and caching corroborated those previously obtained, as we recorded final seed handling rates of over 98% (n = 766). Of these handled acorns, animals removed more than 85% and consumed the rest *in situ* (on the forest surface; Fig. 2). Nearly half of the removed acorns were dispersed (horizontally and/or vertically) by local dispersers, the vast majority by rodents (92.2%, n = 272) and only a small proportion by beetles (7.8%, n = 23; Fig. 2). The rest of the acorns were not relocated and were not used for statistical analyses.

The process of seed deposition differed greatly when the two dispersal agents were compared. In the case of rodents, the destination of the dispersed acorns was strongly affected by the seeds’ microhabitat of origin (Table S1). On the one hand, rodents dispersed acorns mostly to the same type of microhabitat in which they found them (76.1% of the total). Separating by microhabitat types, 100% and 73.2% of the dispersed acorns coming from shrub and tree understory, respectively, remained in the same type of microhabitat, whereas only 43.6% of seeds coming from open sites remained under these conditions. On the other hand, rodents showed a clear preference for those microhabitats located under shrubs when moving acorns from their microsites of origin; 26.8% and 55.1% of the acorns located under trees and in open sites, respectively, were dispersed under shrubs. These changes of microhabitat were less marked in the case of *Q. suber* acorns, as indicated by the significant effect of this factor in the fitted model (Table S1). In summary, rodents dispersed 66.5% of the acorns to shrubs, 20% under trees and 13.5% to open sites (Fig. 2). In contrast, beetles moved very few acorns outside the original microhabitat patch; they displaced them only a few centimetres around the original point and buried most of them in the soil (vertical dispersal). Most of these beetle-handled acorns were located in microhabitats beneath a tree overstory (69.6%), followed by open sites (21.7%) and finally under shrubs (8.7%; Fig. 2).

The distance to which seeds were moved by rodents from their original point was significantly influenced by the microhabitat of origin and the sampling year (Table S2). In 2009/2010, the experimental acorns were dispersed farther than in the 2010/2011 cycle, with longer dispersal distances detected in acorns located beneath trees (1.13±0.37 m, respectively) compared with those located under shrubs (0.84±0.15 m) and in open conditions (0.89±0.31 m). In 2010/2011, the acorns placed in open sites were dispersed farther (1.25±0.19 m) than those protected under shrubs (0.92±0.13 m), but the acorns located under trees were dispersed to shorter distances (0.53±0.10 m) compared with the previous year.

**Figure 3:**
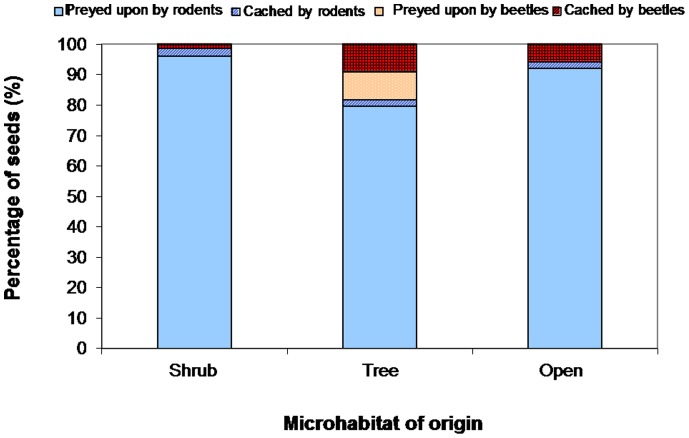
Seed viability after the activity of the two guilds of scatter-hoarding animals evaluated in this study. The percentage of acorns preyed upon (non-viable) or cached and undamaged (viable) by rodents and beetles is represented as a function of the microhabitat of origin (under shrubs, beneath tree canopies and open sites). Percentage values were averaged for the two sampling periods (2009/2010 and 2010/2011) and the two oak species (*Q. suber* and *Q. canariensis*).

The probability of an experimental acorn being preyed upon or undamaged by rodents or beetles (i.e., seed viability) only depended on the microhabitat of origin (Table S3). Thus, the percentage of acorns preyed upon by rodents was higher under shrubs than beneath the tree overstory, with only a small proportion of acorns cached by rodents and beetles (less than 5%) in the most closed microhabitats (Fig. 3). However, the percentage of acorns handled by beetles was much higher in those microhabitats located under trees, with half of them remaining undamaged at the last census (Fig. 3). Acorn fate was not significantly affected by either the sampling year or the oak species (Table S3).

Finally, seed mass did not affect any of the aspects related to the process of seed dispersal that were considered in this study (Tables S1, S2 and S3).

### Seedling Recruitment

**Figure 4:**
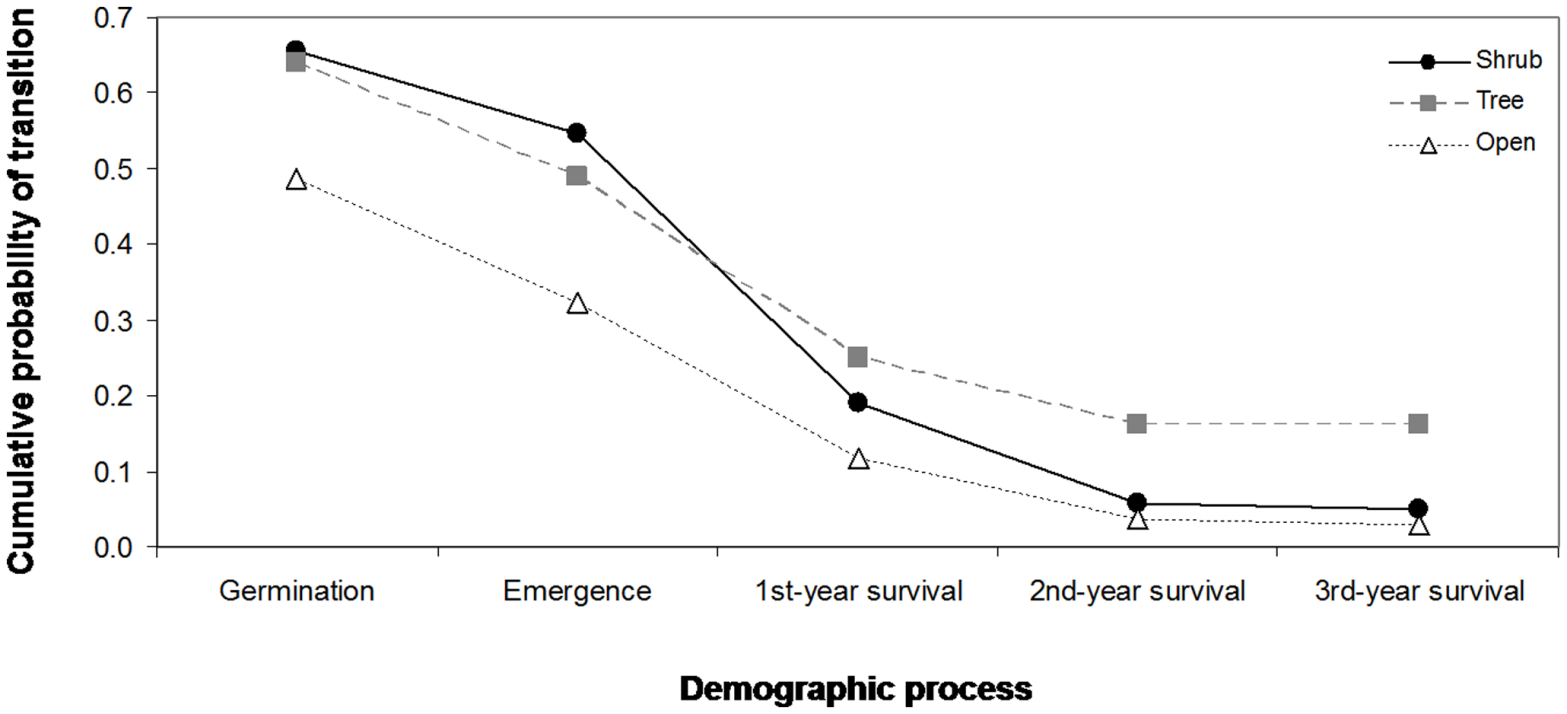
Cumulative probability of transition from seed germination to 3^rd^-year seedling survival in the three microhabitat types. Values of probability were averaged for the two oak species (*Q. suber* and *Q. canariensis*). Microhabitat suitability for each of the demographic processes was assessed by using wire cages with a small mesh size to exclude all potential acorn consumers.

We found significant differences among microhabitats for each of the stage-specific probabilities of recruitment considered in this study. Seed germination and seedling emergence were much lower in open sites than in the other two types of microhabitats (χ = 25.2, p<0.001 and χ = 15.6, p<0.001 for seed germination and seedling emergence, respectively; Fig. 4). Seedling survival was similarly low in open sites and under shrubs, with the highest probabilities of survival in those microhabitats located beneath tree overstories without shrubs (χ = 13.4, p = 0.001 and χ = 24.1, p<0.001 for the first and the second year, respectively; Fig. 4). After three years, seedling survival rate increased strongly and mortality was nearly null in the microhabitats located under trees (χ = 11.5, p<0.001; Fig. 4). As a consequence of these differences among microhabitats, the highest cumulative probability of seedling recruitment was registered under trees (0.16) but similarly low in the other two types of microhabitats (0.03 and 0.05 for open sites and shrubs, respectively, Fig. 4).

### Seed Dispersal Effectiveness

**Figure 5:**
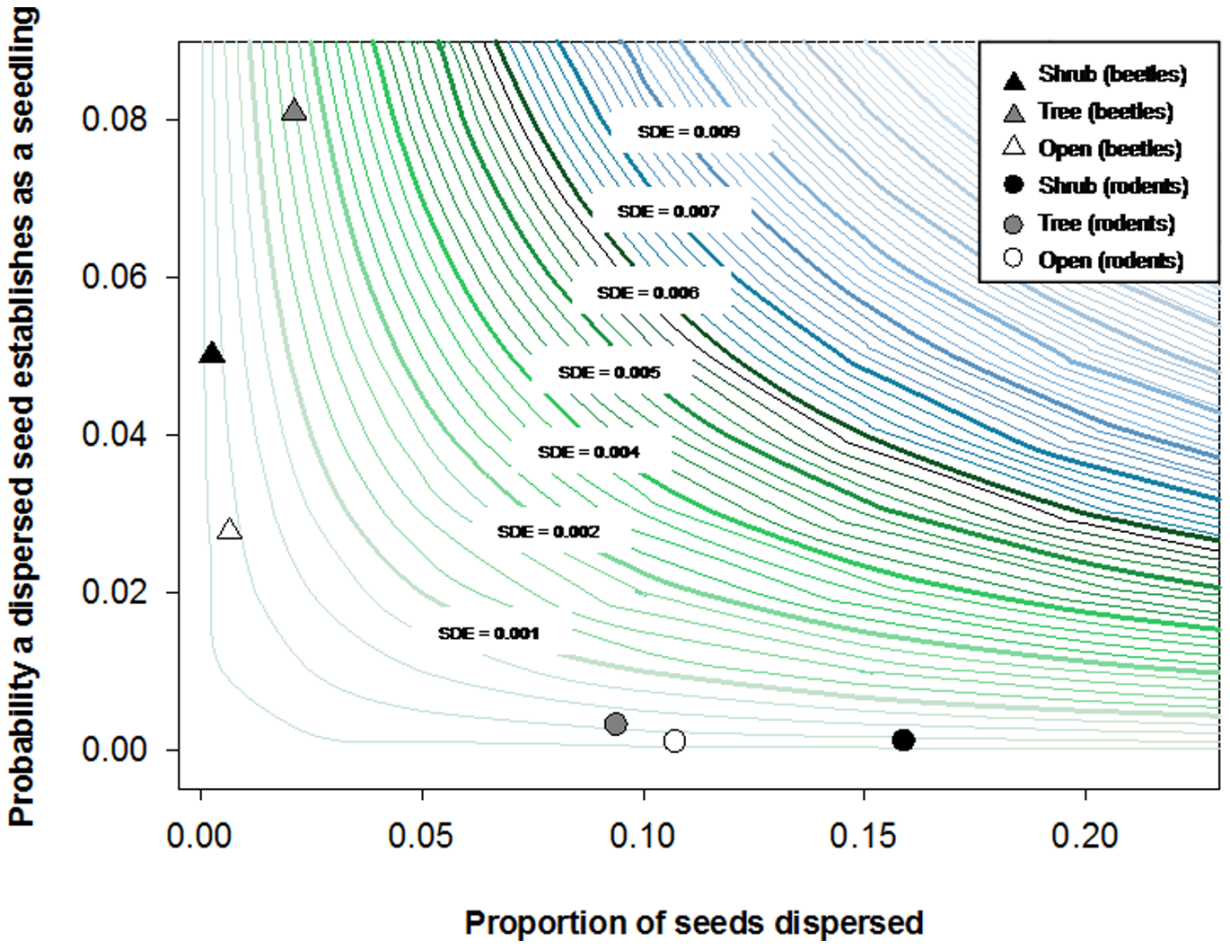
Two-dimensional representation of seed dispersal effectiveness (SDE) landscape. Isoclines represent all combinations of dispersal quantity (*x*-axis) and quality (*y*-axis) that yield the same SDE value. Symbols represent the position on the SDE landscape of the two dispersal agents (rodents represented by circles and beetles by triangles) depending on the microhabitat of origin (shrub in black, tree in grey and open in white colour).

The two dispersal agents compared in this study (rodents and beetles) showed remarkable differences in seed dispersal effectiveness (SDE) due to large variations in the quantitative and qualitative components of SDE. Rodents dispersed a large percentage of acorns of the two oak species, especially under shrubs. However, they consumed more than 95% of these dispersed acorns (265 from a total of 272 dispersed seeds) and only cached a very low proportion of them (less than 3%, n = 7) without signs of embryo damage, resulting in a very low quality of dispersal (Fig. 5). In contrast, beetles handled a comparatively smaller quantity of acorns but they were much more effective than rodents regarding the qualitative component of SDE. Thus, beetles successfully cached (i.e., with the embryo undamaged) a very high proportion of the acorns (15 of a total of 23 dispersed seeds), mostly in microsites located under trees, the microhabitat with the highest quality for seedling recruitment (Fig. 4). The highest SDE value was detected in precisely these microhabitats located under trees (Fig. 5), where the highest rates of seed handling by beetles were registered. In the other two types of microhabitats, however, we found similarly low values of SDE for both beetles and rodents (Fig. 5).

## Discussion

Scatter-hoarding animals (mainly rodents and dung beetles) handled a very large proportion of experimental acorns in the study area (over 98%). This emphasises the seed–seedling transition as a crucial step for the regeneration process of the two oak species studied, as was previously documented for other Mediterranean *Quercus* species [36]–[38]. The estimated rates of seed handling and removal were lower during the first sampling year (2009/2010), likely due to the relatively higher acorn crop size quantified in that year, which led to more abundant food resources for seed consumers on the forest floor. Unexpectedly, scatter-hoarding animals did not show any feeding preference for either of the two oak species, most likely because similar-sized acorns were used in this study for both *Quercus* species. This fact likely mitigated the inter-specific differences in seed removal promoted by variations in seed mass that were previously detected in the study area [39]. Most of the dispersed acorns (over 92%) were due to the action of small rodents. Dung beetles (*Thorectes lusitanicus*) only contributed to a low proportion of acorn dispersal, but with potential repercussions for recruitment and population dynamics for the two study oak species.

### Spatiotemporal Patterns of Seed Dispersal and Recruitment

Spatial patterns of seed dispersal and seedling recruitment were largely explained by the heterogeneity of the forest habitat structure. The microhabitat type where a seed was placed affected both the quantitative component and the two qualitative subcomponents (seed viability and seed deposition) of seed dispersal effectiveness (SDE).

#### Seed handling and viability

The microhabitat of origin strongly determined the quantity of seeds handled and predated by scatter-hoarding animals, highlighting the relevance of habitat structure as mediator of plant-animal interactions in heterogeneous environments [40]. The highest rates of seed handling and predation occurred in those microhabitats located under shrubs, mostly due to the higher foraging activity of rodents. It is well-known that rodents show a clear preference for the most structurally complex microhabitats, where they find more protection against their own predators [39], [41]. However, the probability of being successfully cached (i.e., with the embryo undamaged) was higher in microhabitats located beneath a tree canopy due to the activity of beetles. Previous studies in Mediterranean oak forests have demonstrated that acorn-burier beetle species such as *T. lusitanicus* are more abundant in closed microhabitats compared with open sites [28], [42]. This type of microhabitat most likely attracts a higher number of beetles due to the higher acorn availability on the forest floor and the microclimatic conditions harboured by these habitats. As expected, the highest rates of seed handling and dispersal by beetles were registered in late autumn/early winter, coinciding with the peak of maximum activity of these summer-aestivation insects [30].

#### Seed deposition

The microhabitat distribution of dispersed seeds was largely heterogeneous and mostly promoted by the foraging patterns of rodents. These non-random patterns of seed deposition may have profound implications for the regeneration of both oak species, in accordance with recent studies focused on other Mediterranean *Quercus* species (e.g., [29], [33]). Although rodents tended to disperse most acorns within the same type of microhabitat where they found them, they also moved a relatively important proportion of them from their microsites of origin. Most of these movements between different microhabitats came from acorns placed in open sites, which consequently travelled longer distances. Rodents spent less time in open sites, likely due to the higher risk of predation that they suffer in this type of microhabitat, and preferred to disperse acorns to more protective microsites such as shrubs [43]–[44]. Interestingly, acorns were dispersed farther during the lower-production year, most likely because a higher competition for seed resources favours a less clumped distribution of acorns in order to increase spacing of caches and reduce seed pilfering [33], [45].

#### Seedling recruitment

From the plant’s perspective, it is essential to know whether the different microhabitats in which acorns were dispersed fulfil further requirements for seedling recruitment. Our multi-stage demographic approach allowed us to detect strong differences in microhabitat suitability, which slightly changed over the life-cycle of the plant. Open sites were less suitable for seed germination, seedling emergence and survival owing to the unfavourable environmental conditions generated by events of temporal waterlogging, which are more frequent in this type of microhabitat [32]. The negative effects of excess water on these early life-history stages could be attributed to the slow diffusion rates of gases in water-saturated soils, which hampers oxygen supply to the roots and consequently may impede radicle development and impair seedling survival over the dry summer [46]–[47]. Seedling survival was similarly low in the most closed microhabitats, most likely due to the excess shade provided by the dense canopy of trees and shrubs and the lack of sufficient radiation to fix carbon. As a consequence of these conflicting tendencies through plant ontogeny, microhabitats of intermediate shade (i.e., those located under trees) showed the highest quality for overall recruitment in the short term (until the three-year-old sapling stage), precisely where dung beetles act as effective acorn dispersers (see details below).

The two studied oak species usually form long-living sapling banks under the closed canopy of adults [10] and are able to survive a long time with suppressed growth (‘sit-and-wait’ strategy) until a canopy gap occurs (e.g., after the death of a tree). When exposed to high light conditions, suppressed saplings are able to grow rapidly to reach the canopy layer and potentially colonise these vacant microsites [48]. This strategy is particularly relevant in the study oak forests, where disturbances in canopy cover are occurring at rates much higher than expected as a consequence of one or several threats acting together (i.e., climatic change, diseases and pathogens; [49]). Although the net effect of the tree canopy on recruitment could switch ontogenetically from positive to negative [50], we hypothesise that these microsites of intermediate shade could be used as suitable habitats for oak recruitment in the long term. Nevertheless, further studies on later life-history stages (i.e., juveniles and sub-adults) are necessary to determine whether the estimated high quality of microhabitats located beneath tree overstory will remain invariable throughout the whole life-cycle of the two studied oak species.

### Seed Dispersal Effectiveness: Rodents and Beetles

Rodents and beetles showed remarkable differences in their effectiveness as local acorn dispersers as a consequence of their distinct foraging behaviours and metabolic requirements. Quantitatively, rodents were much more important than beetles because they dispersed the vast majority of acorns. However, they were qualitatively less effective than beetles for two main reasons. First, they consumed most of the dispersed acorns (over 95%), a common feature of disperser assemblages where the main dispersal agents are also major seed consumers [29]. Second, they moved a large proportion of acorns under shrubs, a less suitable microhabitat for seedling survival and thereby for overall recruitment of the two oak species. In contrast, the beetle *T. lusitanicus* dispersed a lower proportion of acorns, but most of them were successfully cached. Thus, a high percentage of these buried acorns were left intact or only partially consumed (most of them with the embryo undamaged), likely due to their lower metabolic requirements compared with rodents. Although beetles only displaced acorns a few centimetres around the original point, they buried nearly all the handled acorns into the soil at a depth (10 cm maximum) compatible with seedling establishment. This type of vertical dispersal could have significant benefits for oak recruitment because burial avoids seed desiccation, stimulates germination and reduces pilferage risk by other seed predators [51]–[52]. In addition, seed caching by beetles occurred predominantly beneath tree overstory – a high-quality microhabitat for overall recruitment (at least in the short term) – mainly because of their marked foraging preferences for this habitat and their restricted ability for pulling back acorns over long distances. As a consequence of these large differences in the qualitative component, the estimated SDE provided by beetles in these microhabitats of intermediate shade was up to ten-fold greater compared with that of rodents.

Therefore, the quality of dispersal was the component that better explained the SDE of scatter-hoarding animals in the Mediterranean oak forests studied and was inversely related to the quantitative component. These results are in accordance with a recent study on a Mediterranean fleshy-fruited species [53], but they contrasted with others reporting positive or negative relationships between both components depending on the habitat [14] or the plant species considered [54]. Thus, the relative importance of each of the two components of SDE for recruitment of a given plant species appears to be influenced by several factors, including the plant-disperser assemblage, the habitat structure, as well as other potential external factors such as food availability for seed consumers or abundance of disperser populations [55]–[56]. Our findings reveal the importance of considering the quality of dispersal for an accurate quantification of SDE and support other studies reporting that the quantitatively most important dispersers are not necessarily the most effective contributors to plant recruitment.

### Implications for Oak Recruitment and Management

The results from this study demonstrate that certain species of dung beetles can act as effective local seed dispersers for some oak species and, thereby, changes in the abundance of their populations could have profound implications for plant recruitment and community dynamics [14]–[15]. Due to their high-quality dispersal, small changes in the quantity of seeds dispersed by beetles (through population increases, for example) could substantially enhance SDE. This hypothetical situation can be easily simulated from the SDE-isocline diagram, where small movements along the *x*-axis could result in large increases in the SDE provided by these high-quality dispersers (Fig. 5). Due to the strong spatial dependence of beetles with dung availability – their main source for nesting and feeding [57] – management policies directed toward controlling the population size of large herbivores may be essential for allowing the persistence and conservation of beetle populations, thus promoting the natural regeneration of *Quercus* species.

Despite their lower effectiveness as local seed dispersers, the potential role of rodents as complementary seed dispersers might be particularly beneficial for oak recruitment in high-production years, where the ‘competitive dispersal’ established between these two guilds of scatter-hoarding animals (i.e., competition between rodents and beetlesas seed dispersal agents) will be most likely less marked [58]. Although we recognise that the rates of successful acorn dispersal by rodents could have been underestimated in this study due to the relatively low seed production quantified in the two sampling years, the estimated values of SDE due to beetles were also most likely underestimated. Thus, in a previous study carried out during a mast year, we detected a very large proportion of acorns (up to 40%) manipulated and buried by beetles, most of them keeping their ability to establish as seedlings [19]. The higher percentage of ‘positive’ burials found for such a year of high seed crop was most likely promoted by a process of seed consumer satiation, enhancing not only the quantity of seeds dispersed by beetles (by reducing competition for resources with rodents) but also the quality of seed dispersal (increasing the probability of an acorn being buried and abandoned later with the embryo undamaged). Nevertheless, further studies in high-production years comparing the effectiveness of rodents and beetles as local seed dispersers are necessary to evaluate the role of these two guilds of scatter-hoarding animals for oak recruitment under a broader range of acorn crop sizes. A multi-species dispersal system including both types of acorn dispersers may be relevant to ensure seedling recruitment and sustain the population equilibrium of Mediterranean *Quercus* species such as the two studied oak species, which have serious regeneration problems in the study area [10], [48]. Understanding and preserving this ‘interaction biodiversity’ (*sensu* [59]) must be considered for improving ecologically based management and restoration strategies in Mediterranean forests.

## Supporting Information

Table S1 **Results from the Generalized Linear Mixed Models testing the different factors (sampling year, oak species and microhabitat of origin) affecting the microhabitat destination of acorns dispersed by rodents.** Seed mass was included in the analysis as a counting covariable. Experimental unit was considered as a random factor. The dependent variable was fitted to a multinomial distribution with three possible categories (shrub, tree or open). Significant factors are highlighted with bold letters.
(TIF)

Table S2 **Results from the Linear Mixed Models evaluating the factors (sampling year, oak species and microhabitat of origin) affecting the dispersal distance (log-transformed) of those experimental acorns dispersed by rodents.** Seed mass was included in the analysis as a counting covariable. Experimental unit was considered as a random factor. Significant factors are highlighted with bold letters.
(TIF)

Table S3 **Results from the Generalized Linear Models analysing the effect of different factors (sampling year, oak species, microhabitat of origin and their interactions) on the final status of experimental acorns (i.e., seed viability).** Seed mass was included in the analysis as a counting covariable. Experimental unit was considered as a random factor. The dependent variable was fitted to a multinomial distribution with four possible categories depending on the combination of dispersal agent (rodents or beetles) and the final seed status (preyed upon versus successfully cached). Significant factors are highlighted with bold letters.
(TIF)
