# Supplementary figures and images for: The Comparative Effectiveness of Rodents and Dung Beetles as Local Seed Dispersers in Mediterranean Oak Forests
Source: PLoS One. 2013 Oct 23;8(10):e77197. doi: 10.1371/journal.pone.0077197 (PMC3806725; doi:10.1371/journal.pone.0077197)

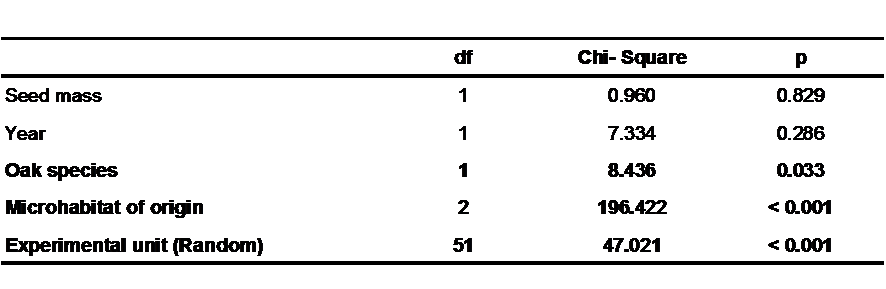

Supplement: Table S1 — Results from the Generalized Linear Mixed Models testing the different factors (sampling year, oak species and microhabitat of origin) affecting the microhabitat destination of acorns dispersed by rodents. Seed mass was included in the analysis as a counting covariable. Experimental unit was considered as a random factor. The dependent variable was fitted to a multinomial distribution with three possible categories (shrub, tree or open). Significant factors are highlighted with bold letters. (TIF) [file pone.0077197.s001.tif]

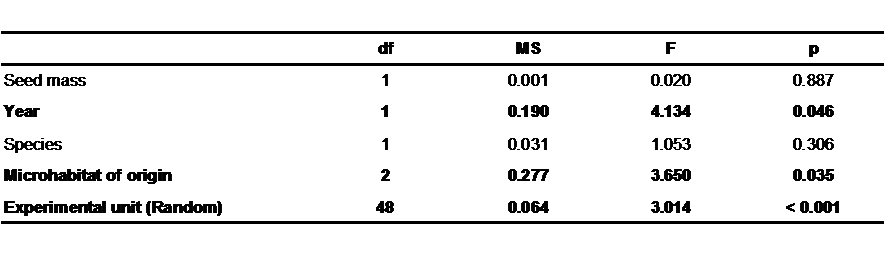

Supplement: Table S2 — Results from the Linear Mixed Models evaluating the factors (sampling year, oak species and microhabitat of origin) affecting the dispersal distance (log-transformed) of those experimental acorns dispersed by rodents. Seed mass was included in the analysis as a counting covariable. Experimental unit was considered as a random factor. Significant factors are highlighted with bold letters. (TIF) [file pone.0077197.s002.tif]

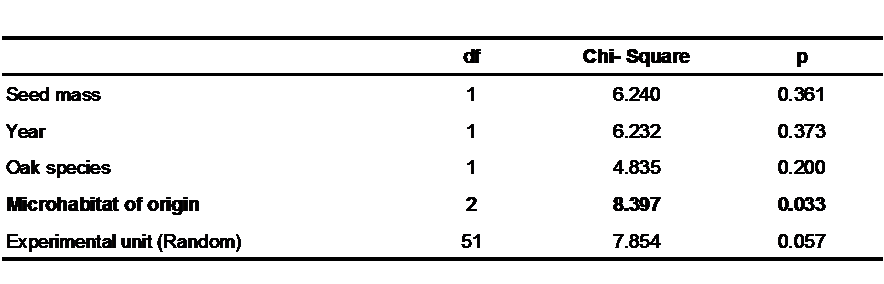

Supplement: Table S3 — Results from the Generalized Linear Models analysing the effect of different factors (sampling year, oak species, microhabitat of origin and their interactions) on the final status of experimental acorns (i.e., seed viability). Seed mass was included in the analysis as a counting covariable. Experimental unit was considered as a random factor. The dependent variable was fitted to a multinomial distribution with four possible categories depending on the combination of dispersal agent (rodents or beetles) and the final seed status (preyed upon versus successfully cached). Significant factors are highlighted with bold letters. (TIF) [file pone.0077197.s003.tif]
